# Comparison of tegoprazan-based and proton pump inhibitor-based regimens for *Helicobacter pylori* eradication: a meta-analysis and systematic review

**DOI:** 10.3389/fmed.2025.1580203

**Published:** 2025-06-18

**Authors:** Xin Zhang, Xing Li, Jiangguo Li, Yuexia Deng, Wei Xu, Dongkui Chen, Licheng Wei

**Affiliations:** ^1^Department of Gastroenterology, The Fourth Hospital of Changsha (Integrated Traditional Chinese and Western Medicine Hospital of Changsha), Changsha Hospital of Hunan Normal University, Changsha, China; ^2^Department of Critical Care Medicine, Changsha Hospital of Traditional Chinese Medicine (Changsha No. 8 Hospital), Changsha, China

**Keywords:** *Helicobacter pylori*, tegoprazan, proton pump inhibitor, eradication, compliance, adverse events

## Abstract

**Background and aim:**

Tegoprazan (TEG) is a novel potassium-competitive acid blocker (P-CAB) that provides long-lasting acid-suppressing effects. The role of TEG-based *Helicobacter pylori* (*H. pylori*) eradication regimens in comparison to proton pump inhibitor (PPI)-based regimens requires further investigation.

**Methods:**

We conducted a comprehensive search across multiple databases. Studies comparing *H. pylori* eradication rates, adverse events (AEs), and compliance between TEG-based and PPI-based regimens were included. Statistical analyses were performed using RevMan 5.4.

**Results:**

A total of eight studies involving 4,640 patients were included. Based on intention-to-treat (ITT) analyses, the overall eradication rate (78.6% vs. 76.6%; odds ratio [OR] = 1.08, 95% CI: 0.93–1.24; *p* = 0.31, *I*^2^ = 0%) and compliance (97.8% vs. 97.8%; OR = 1.16, 95% CI: 0.54–2.50; *p* = 0.33, *I*^2^ = 13%) were comparable between the TEG and PPI groups. The AE rate of TEG-based regimens was significantly lower than that of PPI-based regimens (30.8% vs. 34.9%; OR = 0.88; 95% CI: 0.78–1.00; *p* = 0.04, *I*^2^ = 67%), although this difference was not significant in the randomized controlled trials (RCTs). The subgroup analyses showed higher eradication rates in studies conducted in China, those with treatment durations of 10 or 14 days, and those using dual or bismuth quadruple regimens. However, the treatment regimens did not significantly influence eradication rates within any subgroup.

**Conclusion:**

TEG-based *H. pylori* eradication treatment demonstrated similar eradication rates, compliance, and safety to PPI-based regimens.

**Systematic review registration:**

https://www.crd.york.ac.uk/PROSPERO/view/CRD42024629665.

## 1 Introduction

More than half of the people worldwide have been infected with *Helicobacter pylori* (*H. pylori*) (1, 2). *H. pylori* is a known human carcinogen that is strongly associated with the development of gastric cancer (GC), peptic ulcers, and B-cell mucosa-associated lymphoid tissue lymphoma (3). Many gastrointestinal disorders, including GC, can be prevented and treated through the eradication of *H. pylori* (2, 4, 5). Therefore, administering *H. pylori* eradication therapy to infected individuals holds significant clinical importance.

For first-line *H. pylori* eradication regimens, proton pump inhibitor (PPI)-based triple or quadruple therapies are recommended in different countries (6–8). However, the efficacy of these regimens has declined significantly in recent years, primarily due to antibiotic resistance (9, 10) and insufficient acid suppression (11, 12). Increasing intragastric pH levels may enhance the stability and concentration of antibiotics, such as amoxicillin and clarithromycin (13). Furthermore, sustained acid suppression could render *H. pylori* more susceptible to antibiotic-induced eradication (12, 14). However, PPIs have limitations, including a short half-life, delayed onset of action, and variability in efficacy influenced by dietary factors and CYP2C19 polymorphisms (15). Therefore, identifying and utilizing more effective acid inhibitors in *H. pylori* eradication therapy is critical.

The recently developed potassium-competitive acid blocker (P-CAB) class provides potent acid suppression (16). The first developed P-CAB, vonoprazan, has demonstrated similar or superior efficacy in *H. pylori* eradication treatment, as shown by several meta-analyses (17–20). Tegoprazan (TEG), a subsequent P-CAB, has shown comparable or even superior acid suppression compared to vonoprazan or PPIs (21). In addition to acid suppression, P-CABs may also increase the susceptibility of antibiotic-resistant *H. pylori* strains (22). While these properties suggest promising eradication outcomes, evidence remains limited and inconsistent. Additionally, the optimal drug combinations and treatment duration for TEG-based regimens remain undefined. A comprehensive comparative analysis of TEG-based versus PPI-based regimens is therefore warranted to establish optimal eradication strategies.

This study aimed to evaluate the relative safety and efficacy of TEG-based versus PPI-based regimens for *H. pylori* infection through a systematic review and meta-analysis.

## 2 Methods

The meta-analysis was performed according to the guidelines of the Preferred Reporting Items for Systematic Reviews and Meta-Analyses (PRISMA) and the Cochrane Handbook for Systematic Reviews of Interventions, as described in previous meta-analyses (23, 24). We prospectively registered this meta-analysis on PROSPERO (CRD42024629665). The approval from the Ethics Committee was waived due to the study being a systematic review and meta-analysis.

### 2.1 Search strategy

To identify relevant English-language studies, two independent researchers conducted comprehensive searches of PubMed, Embase, Web of Science, and the Cochrane Library (updated to 20 December 2024). The search strategy included terms related to TEG, eradication, and *H. pylori*. Detailed search terms for PubMed are provided in Supplementary Table 1.

### 2.2 Study selection

All articles were assessed by two independent researchers to decide which ones could be included. In case of disagreement, the corresponding author (L.X) was consulted to make the final decision. The inclusion criteria were as follows: (1) patients: diagnosed with *H. pylori* infection; (2) intervention: TEG-based *H. pylori* eradication regimens for first-line therapy; (3) comparison: eradication therapy with PPI-based regimens; (4) outcomes: successful eradication rate and adverse event (AE) rate; and (5) study design: randomized controlled trial (RCT) or retrospective case–control study.

### 2.3 Data extraction

Two researchers independently extracted the following data: first author, publication year, country, patient age, diagnostic method, treatment regimen details, sample size, eradication success rate, and adverse events.

### 2.4 Risk of bias assessment

The Cochrane risk-of-bias (ROB) tool 2.0 and the Newcastle–Ottawa Scale (NOS) were used to assess bias in RCTs and retrospective studies, respectively. Assessments were performed independently by two researchers (Z.X. and L.J.G.).

### 2.5 Statistical analysis

Primary endpoints included the eradication rate and AE rate based on intention-to-treat (ITT) analysis. Only drug-related AEs were recorded, with serious AEs analyzed separately. Pooled proportions were calculated using RevMan 5.4, with heterogeneity assessed using the I^2^ statistic. A random-effects model was applied if I^2^ > 50%; otherwise, a fixed-effects model was used. Publication bias was evaluated using funnel plot visualization.

Sensitivity analysis was performed by sequentially excluding individual studies to assess their impact on the overall results. Subgroup analyses explored heterogeneity based on treatment regimen (high-dose dual therapy vs. triple therapy vs. bismuth quadruple therapy) and duration (7 vs. 10 vs. 14 days).

The results were reported as odds ratios (ORs) with 95% confidence intervals (CIs). A *p*-value < 0.05 was considered statistically significant.

## 3 Results

### 3.1 Search results and characteristics of the included studies

**Figure 1:**
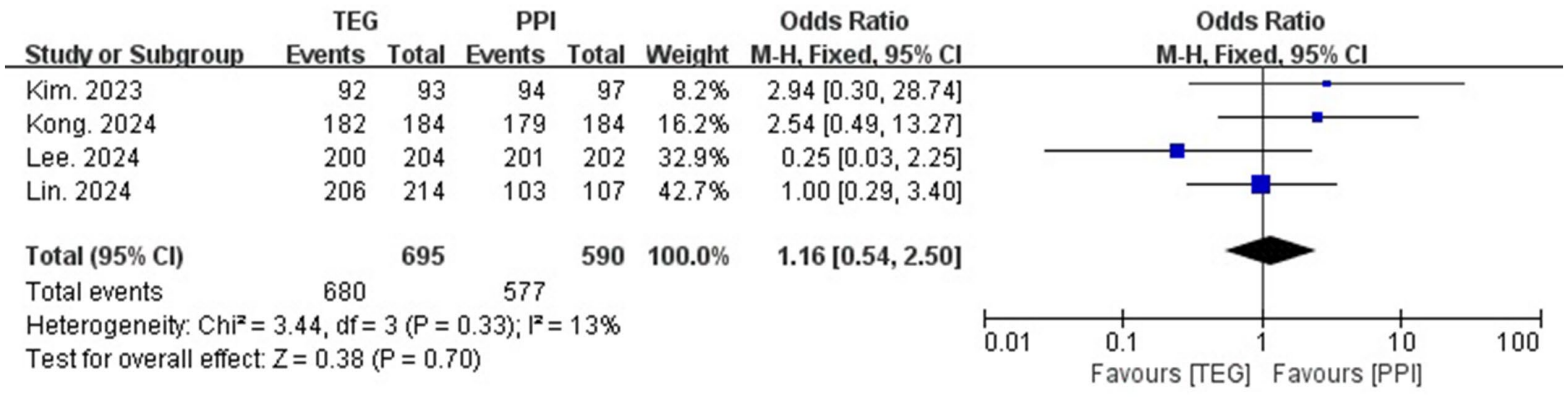
Search flow diagram in accordance with the preferred reporting items for systematic reviews and meta-analyses principles.

Initially, 36 studies from different databases were screened. Based on the inclusion and exclusion criteria, eight studies were finally included (25–32). Figure 1 provides a summary of the study selection flow diagram.

Table 1 displays the features of the included studies. They were published between 2022 and 2024. A total of six studies were conducted in Korea, and two were conducted in China. In addition, five studies were RCTs, and three were retrospective studies. All patients in these eight studies were treatment-naïve. All doses of TEG were 50 mg twice daily, except for one arm in the study by Lin et al. (31) (50 mg once daily, *n* = 107). For dual treatment, the antibiotic used was amoxicillin at a dosage of 3 g/day. For triple treatment, the antibiotics used were amoxicillin at a dosage of 1 g and clarithromycin at a dosage of 500 mg twice daily. The bismuth-based triple treatment was combined with the quadruple therapy. Particularly, the study by Jung et al. (29) mentioned concomitant therapies, with one acid inhibition regimen combined with three antibiotics. Another study by Lee et al. (30) described sequential therapies, with a change of antibiotics after 5 days of treatment. The majority of studies (5/8) included a treatment duration of 14 days, two studies had a duration of 10 days, and the other study had a duration of 7 days.

**Table 1:** Characteristics and main results of included studies in this meta-analysis.

Study	Country	Study design	Treatment experience	Sample size (T/P)	Treatment regimens for TEG group	Treatment regimens for PPI group	Treatment duration	Successful eradication rate (T/P), %	Compliance, (T/P), %	Adverse event rate (T/P), %	Severe adverse event (T/P), n
Choi et al. (25)	Korea	Multicenter RCT	First	175/175	Tegoprazan-based triple therapy	Lansoprazole-based triple therapy	7 days	62.86/60.57	NR	37.79/33.53	0/2
Jung et al. (26)	Korea	Multicenter Retrospective	First	344/333	Tegoprazan-based triple therapy	Rabeprazole-based triple therapy	14 days	76.7/75.4	NR	27.6/25.8	NR
Kim et al. (27)	Korea	Multicenter RCT	First	93/97	Tegoprazan-based bismuth quadruple therapy	Lansoprazole-based bismuth quadruple therapy	14 days	90.3/84.5	99.1/97.0	39.1/43.4	1/2
Kong et al. (28)	China	Multicenter RCT	First	184/184	Tegoprazan-based dual therapy	Esomeprazole-based dual therapy	14 days	85.8/84.2	98.9/97.2	16.3/21.2	0/0
Jung et al. (29)	Korea	Multicenter Retrospective	First	620/854	Tegoprazan combined with amoxicillin, clarithromycin, and metronidazole	Rabeprazole combined with amoxicillin, clarithromycin, and metronidazole	10 days	74.7/72.7	NR	39.2/40.6	1/3
Lee et al. (30)	Korea	Single-center RCT	First or experienced	204/202^#^	Tegoprazan combined with amoxicillin for 5 days, followed by tegoprazan combined with clarithromycin and metronidazole for the next 5 days	Esomeprazole combined with amoxicillin for 5 days, followed by esomeprazole combined with clarithromycin and metronidazole for the next 5 days	10 days	87.1/83.8	98.0/99.5	37.1/30.4	21/11
Lin et al. (31)	China	Multicenter RCT	First	214^*^/107	Arm one: tegoprazan-based dual therapy (50 mg twice daily) arm two: tegoprazan-based dual therapy (50 mg once daily)	Esomeprazole-based bismuth quadruple therapy	14 days	85.98 and 85.98/85.05	96.2/96.2	14.56/13.33/27.18	1 and 0/ 1
Park et al. (32)	Korea	Single-center Retrospective	First	435/419	Tegoprazan-based triple therapy	Esomeprazole/sodium bicarbonate-based triple therapy	14 days	78.6/81.4	NR	28.0/39.4	0/0

^#^There were 7 and 15 patients in TEG and PPI groups had previous eradication failure, respectively. *107 patients received tegoprazan 50 mg twice daily and 107 patients received tegoprazan 50 mg once daily. RCT: randomized controlled trial; TEG: tegoprazan; PPI: proton pump inhibitor; T/P: tegoprazan-based group/ PPI-based group, NR: not reported.

### 3.2 Risk of bias for the included studies

All three retrospective studies were of high quality according to the NOS scale (Supplementary Table 2). For the remaining five RCTs, blinding of participants and personnel was the main source of potential bias according to the ROB tool 2.0. The double-blinding method was not used in two studies (Supplementary Figure 1). The study by Lin et al. had a moderate risk of bias, as it did not describe the randomization method in detail, and the blinding method was not used in this study (31).

### 3.3 *Helicobacter pylori* eradication rate

#### 3.3.1 Overall eradication rate

**Figure 2:**
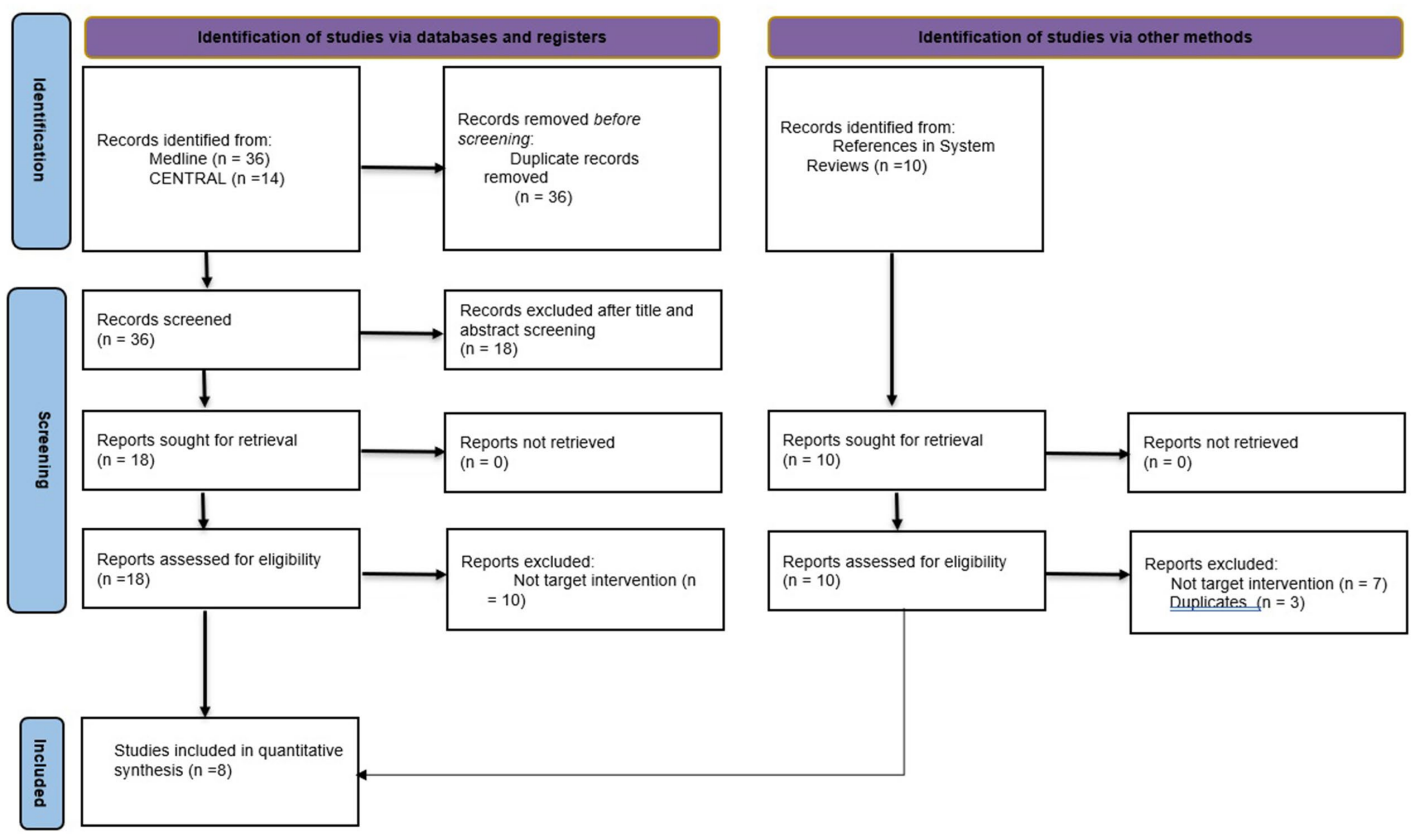
Forest plots comparing the overall eradication rate of *H. pylori* between TEG-based therapy vs. PPI-based therapy.

A total of 2,269 and 2,371 patients underwent *H. pylori* eradication treatment in the TEG and PPI groups, respectively. The overall eradication rates were 78.6% (TEG) and 76.6% (PPI), with no statistically significant difference between the two groups (OR = 1.08, 95% CI: 0.93–1.24; *p* = 0.31; I^2^ = 0%) (Figure 2). Similarly, no significant differences were observed in RCTs (82.1% vs. 78.8%; OR = 1.20, 95% CI: 0.93–1.54; *p* = 0.17; I^2^ = 0%) or retrospective studies (76.4% vs. 75.5%; OR = 1.03, 95% CI: 0.87–1.21; *p* = 0.77; I^2^ = 0%) (Figure 2).

#### 3.3.2 The impact of regions on the overall eradication rate

**Figure 3:**
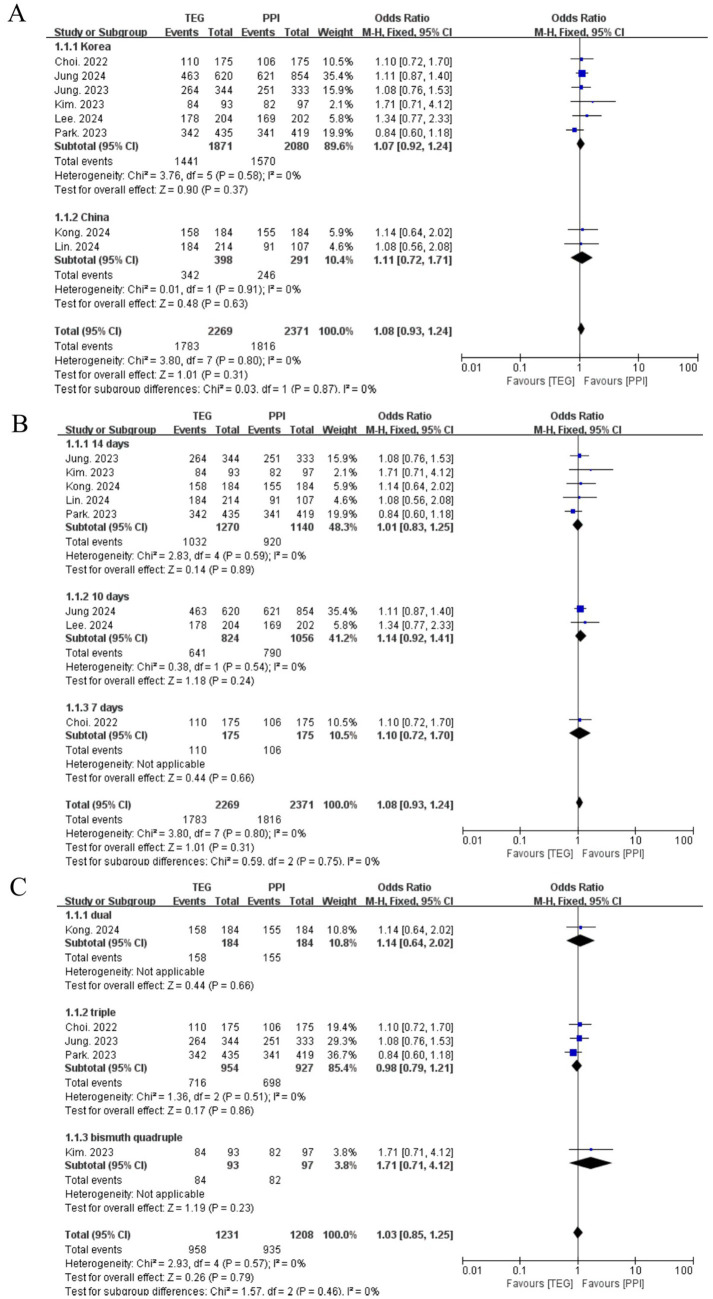
Forest plots comparing the *H. pylori* eradication rate between TEG-based therapy and PPI-based therapy by region (Korea vs. China) **(A)**, length of treatment (7-,10- and 14- days) **(B)**, and treatment regimens (bismuth-containing quadruple therapy, bismuth-containing triple therapy, and non-bismuth-containing dual therapy) **(C)**.

Due to regional variations in antibiotic resistance and CYP2C19 polymorphism distribution, the subgroup analyses were stratified by country. The overall eradication rate was significantly higher in studies conducted in China (85.3%) compared to Korea (76.2%) (*p* < 0.001) (Table 2). However, the TEG-based regimens showed no superiority over the PPI-based regimens in both countries. In Korea, the success rates were 77.0% for TEG-based regimens compared to 75.5% for PPI-based regimens (OR = 1.07, 95% CI: 0.92–1.24; *p* = 0.58; I^2^ = 0%). In China, the success rates were 78.6% for TEG-based regimens compared to 76.6% for PPI-based regimens (OR = 1.11, 95% CI: 0.72–1.71; *p* = 0.91; I^2^ = 0%) (Figure 3A).

**Table 2:** The impact of regions, treatment length, and treatment regimens on eradication rate.

Regions	Total patients, *n*	Successfully eradicated, *n*	Eradication rate, %	*p* value
Korea	3,951	3,011	76.2	<0.001
China	689	588	85.3	
Treatment length				
14 days	2,410	1952	81.0	<0.001^a^
10 days	1880	1,431	76.1	<0.001^b^
7 days	350	216	61.7	<0.001^c^
Treatment regimens				
Dual	475	405	85.3	<0.001^d^
Triple	1881	1,414	75.2	0.624^e^
Bismuth quadruple	297	257	86.5	<0.001^f^

a 14 days vs. 10 days.

b 14 days vs. 7 days.

c 10 days vs. 7 days.

d Dual vs. triple.

e Dual vs. Bismuth quadruple.

f Triple vs. Bismuth quadruple.

#### 3.3.3 The impact of treatment length on the overall eradication rate

The length of treatment was analyzed. In total, 350 patients received 7-day eradication treatment, 1,880 patients received 10-day eradication treatment, and 2,410 patients received 14-day eradication treatment. The eradication rates for the 10-day and 14-day treatments were significantly higher than those for the 7-day treatment (76.1% vs. 61.7%, *p* < 0.001, and 81.0% vs. 61.7%, *p* < 0.001). Moreover, the 14-day treatment showed a superior eradication rate than the 10-day treatment (81.0% vs. 76.1%, *p* < 0.001) (Table 2).

The TEG-based treatment showed no difference when compared to the PPI-based treatment for 14-day (81.3% vs. 80.7%, OR = 1.01, 95% CI:0.83–1.25; *p* = 0.59, I^2^ = 0%), 10-day (77.8% vs. 74.8%, OR = 1.14, 95% CI:0.92–1.41; *p* = 0.54, I^2^ = 0%) and 7-day eradication treatments (62.9% vs. 60.6%, OR = 1.10, 95% CI:0.72–1.70; *p* = 0.66) (Figure 3B).

#### 3.3.4 The impact of treatment regimens on the overall eradication rate

The study by Lee et al. (30) used sequential eradication treatment, while the study by Jung et al. used a three-antibiotic regimen combined with acid suppression, both of which were different from other studies. Therefore, these two studies were excluded from the subgroup analysis. Moreover, one arm in the study by Lin et al. (31) used TEG once daily for eradication treatment, and the arm was excluded from the subgroup analysis.

In total, 475, 1,881, and 297 patients received dual, triple, and bismuth quadruple treatments, with eradication rates of 85.3, 75.2, and 86.5%, respectively. The eradication rates for dual and bismuth quadruple treatments were significantly higher compared to the triple treatment (both *p* < 0.001) (Table 2).

The TEG-based treatment showed no superiority over the PPI-based treatment among the dual (85.7% vs. 84.2%, OR = 1.14, 95% CI:0.64–2.02; *p* = 0.66), triple (75.1% vs. 75.3%, OR = 0.98, 95% CI:0.79–1.21; *p* = 0.86, I^2^ = 0%), and bismuth quadruple treatments (90.3% vs. 84.5%, OR = 1.71, 95% CI:0.71–4.12; *p* = 0.23) (Figure 3C).

### 3.4 Compliance

**Figure 4:**
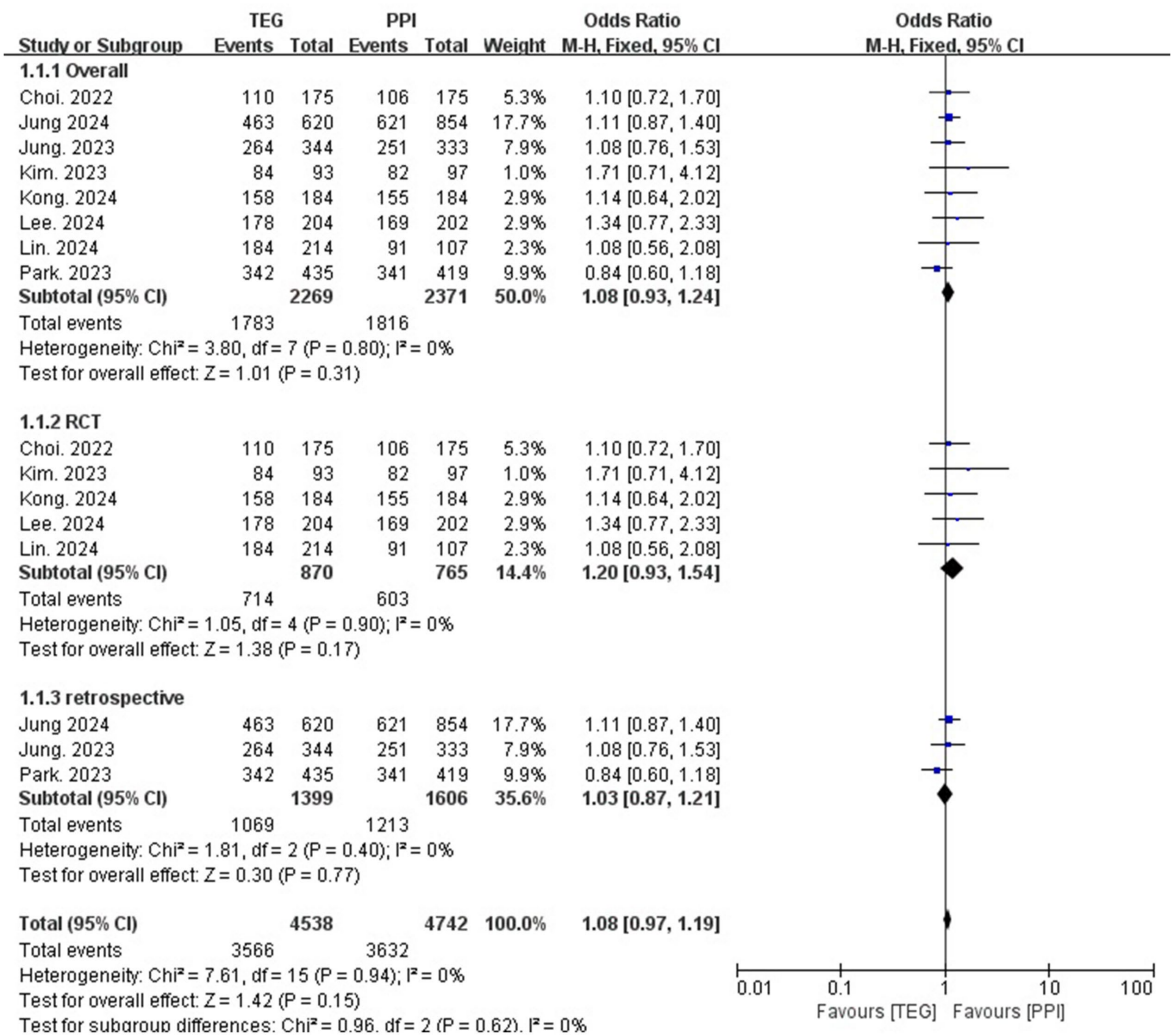
Forest plots comparing TEG-based therapy and PPI-based therapy on compliance.

In total, four studies reported patient compliance. Both groups showed high compliance (97.8% vs. 97.8%, OR = 1.16, 95% CI:0.54–2.50; *p* = 0.33, I^2^ = 13%) (Figure 4).

### 3.5 Adverse events

**Figure 5:**
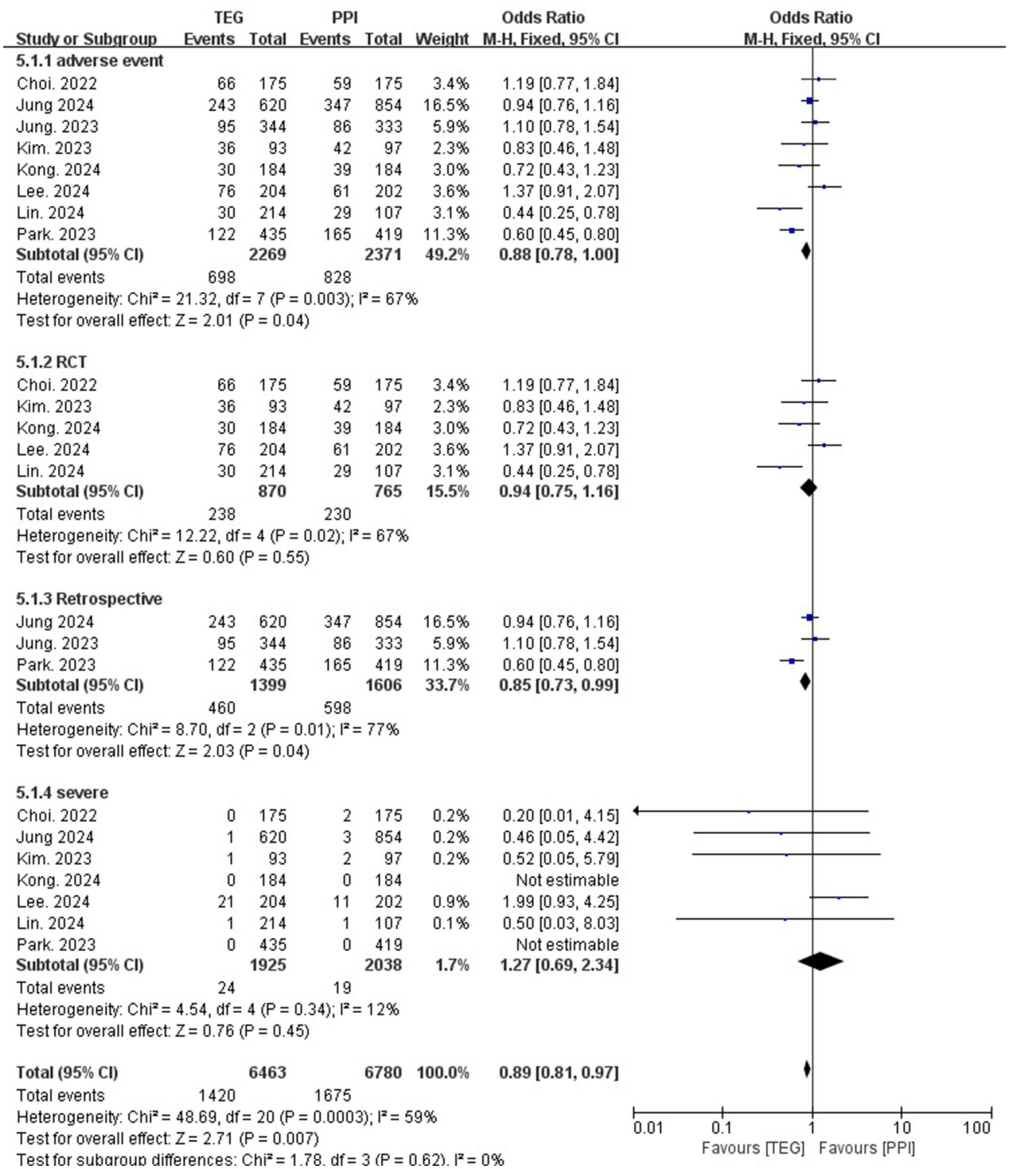
Forest plots comparing TEG-based therapy and PPI-based therapy on overall adverse events and severe adverse events.

The overall AE rate of the TEG-based regimens was significantly lower than that of the PPI-based regimens (30.8% vs. 34.9%; OR = 0.88; 95% CI: 0.78–1.00; *p* = 0.04, I^2^ = 67%) (Figure 5). However, the difference was not obvious among the RCTs (27.4% vs. 30.4%, OR = 0.94; 95% CI: 0.75–1.16; *p* = 0.55, I^2^ = 67%) (Figure 5). For severe adverse event rates, no significant difference was identified (1.2% vs. 0.9%, OR = 1.27; 95% CI: 0.69–2.34; *p* = 0.45, I^2^ = 12%) (Figure 5).

### 3.6 Publication bias and sensitivity analysis

The funnel plot results illustrated that the eradication rate was essentially symmetrical, suggesting that this study did not experience significant publication bias (Supplementary Figure 2). Moreover, the research findings were rather solid since the sensitivity analysis did not significantly alter the results of the overall *H. pylori* eradication rate.

## 4 Discussion

In this systematic review and meta-analysis, we comprehensively searched databases to extract the published studies that compared TEG-based regimens with PPI-based regimens in the eradication treatment of *H. pylori*. The results showed that the TEG-based treatment had a comparable eradication rate and compliance with the PPI-based therapy in the ITT analysis. However, the overall AE rate was significantly lower for the TEG-based treatment, although the difference was not obvious in RCTs.

TEG was first approved in 2018 in South Korea. The drug showed promising advantages in the treatment of acid-associated diseases. When administered at 50 mg once daily, symptom relief rates reached 86.7% at 2 months for the treatment of functional dyspepsia (33). TEG 50 mg administered once daily is comparable to lansoprazole 30 mg taken once daily in Chinese patients suffering from duodenal ulcers (34). Similarly, when compared to esomeprazole once daily, TEG 50 mg once daily showed comparable tolerability and non-inferior effectiveness in healing erosive esophagitis, improving symptoms, and enhancing quality of life (35). Evidence supporting the use of TEG in *H. pylori* eradication treatment has accumulated in recent years. In 2024 and 2025, two meta-analyses by Kanu et al. (36) and Cho et al. (37) reached the same conclusions as our meta-analysis. However, the lack of subgroup analysis and the smaller number of included studies restricted the reliability of these meta-analyses. Therefore, we conducted a meta-analysis with the most recent evidence to examine how TEG-based regimens may be used to treat *H. pylori* infection.

Antibiotics, probiotics, and bismuth were the mainstays of previous treatment regimen modifications; nonetheless, the effectiveness of *H. pylori* eradication has continued to decline. Currently, selecting acid-suppressive medications presents another opportunity for therapeutic innovation. Acid suppressive agents lower the minimum inhibitory concentration (MIC) of medications, which is essential for the sterilization process, while also creating the proper pH environment for antibiotic sterilization (1). Long-term acid suppression, particularly at night, is essential for eliminating *H. pylori* (37, 38). Compared to patients who tested positive for nocturnal acid breakthrough (NAB), those without NAB had a higher eradication rate (38). P-CABs have been proven to be more effective in inhibiting gastric acid secretion compared to traditional PPIs (39). The first-generation P-CAB, vonoprazan, showed at least non-inferior efficacy and safety compared to conventional PPI-based therapies for both naive patients and patients with previous treatment failure (17, 19). However, the efficacy of TEG-based treatment was not verified in this study. Head-to-head comparisons between vonoprazan and TEG are needed to clarify the results. However, pharmacological studies are limited. The effects of TEG, vonoprazan, and esomeprazole on acid suppression at night in healthy volunteers were compared in a recent study (21). Compared to vonoprazan and esomeprazole, TEG inhibited nocturnal acid production more rapidly when administered at bedtime. However, vonoprazan caused greater and longer-lasting increases in intragastric pH over time. Moreover, P-CABs appear to induce varying degrees of hypergastrinemia, with vonoprazan causing a more significant increase compared to TGE, despite both having nearly identical antisecretory effects (39). Therefore, vonoprazan may have longer-lasting acid suppression effects than TEG, and it may be the reason why TEG-based therapies are not superior to PPI-based therapies. Moreover, as TEG suppresses gastric acid secretion at 50, 100, 200, and 400 mg in a dose-dependent manner (40), higher doses of TPZ may be considered for *H. pylori* treatment in the future.

The incidence of AEs appeared to be lower in patients who received TEG-based treatment. The most commonly reported adverse events in the two treatment regimens were abdominal pain, abdominal bloating, diarrhea, headache, and dysgeusia. The self-reported nature of these symptoms may explain the high heterogeneity observed in the analysis of adverse events between the two groups. The majority of AEs were well-tolerated, transient, and did not require any medical intervention. As a result, the incidences of severe AEs and compliance in two treatment groups did not differ significantly. Given that the *p*-value was close to the cutoff (*p* = 0.04) and the difference was not obvious in the RCTs, either treatment regimen was feasible. The advantage of TEG lies in the convenience of medication, as the efficacy of the drug is not influenced by diet (41).

According to the subgroup analysis, the studies conducted in China with a treatment duration of 10–14 days and using dual or bismuth quadruple treatments showed higher eradication rates compared to the studies conducted in Korea with a 7-day treatment duration and triple treatments. These results are consistent with those of previous studies. In a European RCT, 307 patients were randomized to receive 7-day, 10-day, or 14-day triple therapy. Both the 10-day and 14-day regimens reached eradication rates above the threshold of 80% in the ITT analysis (42). Another meta-analysis by Ding et al. (43) found that 14-day and 10-day bismuth-containing quadruple regimens had similar efficacy and a lower incidence of adverse effects. A network meta-analysis concluded that quadruple and high-dose dual therapies achieved identical eradication rates compared to standard triple therapy (44). In the studies conducted in Korea, triple treatment was the most frequently used regimen, and one study used a 7-day treatment duration. As a result, the overall eradication rate was significantly lower in Korea. More aggressive strategies should be implemented in Korea to ensure the efficacy of eradication treatment.

In our study, only the results from the ITT analysis were analyzed. As mentioned earlier, the treatment regimens were well-tolerated, and the results of the ITT analysis were comparable to those of the pre-protocol analysis. Moreover, it is important for patients to closely follow the instructions of the eradication regimen. PPI should be taken on an empty stomach, while TEG is not affected by dietary intake (45, 46). The difficulty in appropriately using the drugs was not balanced between the two groups. Therefore, the ITT analysis may truly reflect the treatment efficacy for different regimens.

There are several limitations to this study. First, the fact that all of the included studies were from East Asia might have limited the generalizability of the research findings. Second, there was variation in the antibiotics and PPIs used across the studies. This could have introduced bias in the efficacy and safety analyses. Third, the number of studies and sample sizes for the subgroup analysis were limited.

## 5 Conclusion

The TEG-based *H. pylori* eradication therapy demonstrated comparable efficacy (eradication rate) and compliance to the PPI-based regimens in the ITT analysis. While the overall AE rate was significantly lower in the TEG-based treatment, this difference was not statistically significant in RCTs. The findings from this meta-analysis suggest that TEG-based regimens represent a viable alternative for *H. pylori* eradication therapy.

## Data Availability

The raw data supporting the conclusions of this article will be made available by the authors, without undue reservation.

## Supplementary material

SUPPLEMENTARY FIGURE 1 Assessment of bias risk for the included randomized controlled studies based on Cochrane risk of bias tool 2.0.

SUPPLEMENTARY FIGURE 2 Funnel plot of the overall eradication rate.
