# Supplementary figures and images for: Comparison of tegoprazan-based and proton pump inhibitor-based regimens for Helicobacter pylori eradication: a meta-analysis and systematic review
Source: Front Med (Lausanne). 2025 Jun 18;12:1580203. doi: 10.3389/fmed.2025.1580203 (PMC12215699; doi:10.3389/fmed.2025.1580203)

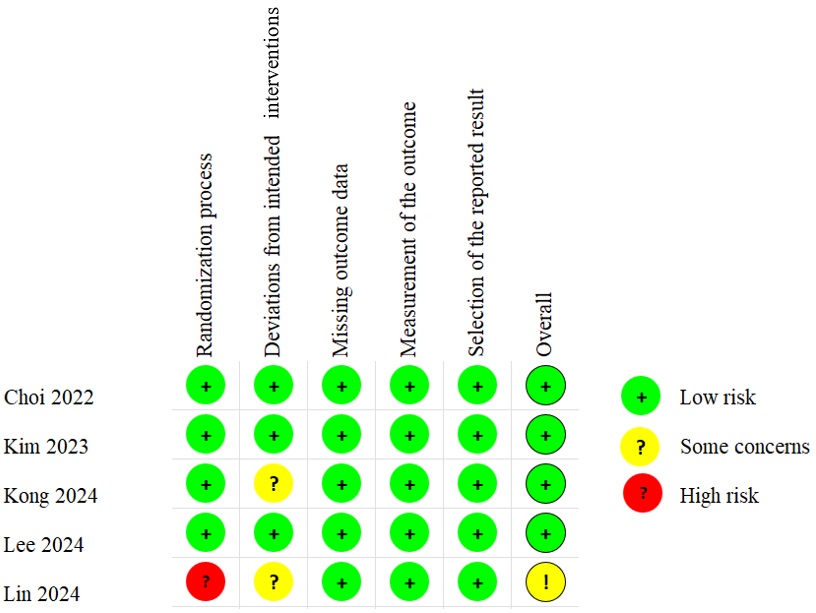

Supplement: SUPPLEMENTARY FIGURE 1 — Assessment of bias risk for the included randomized controlled studies based on Cochrane risk of bias tool 2.0. [file Image_1.jpeg]

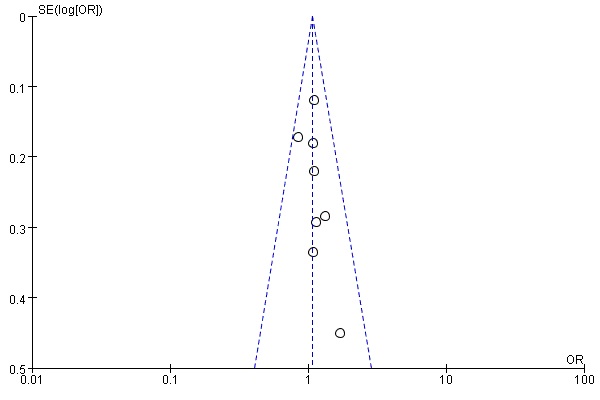

Supplement: SUPPLEMENTARY FIGURE 2 — Funnel plot of the overall eradication rate. [file Image_2.jpeg]
