# Supplementary material for: Comparison of tegoprazan-based and proton pump inhibitor-based regimens for Helicobacter pylori eradication: a meta-analysis and systematic review
Source: Front Med (Lausanne). 2025 Jun 18;12:1580203. doi: 10.3389/fmed.2025.1580203 (PMC12215699; doi:10.3389/fmed.2025.1580203)
Supplement: Supplementary file 3 [file Table_1.docx]

**Supplementary Table 1** Quality evaluations of all the included articles according to the Newcastle–Ottawa scale

| **Study** | **Year** |  | **Selection** | | | |  | **Comparability** | |  | **Outcome** | | |  | **Total score** |
| --- | --- | --- | --- | --- | --- | --- | --- | --- | --- | --- | --- | --- | --- | --- | --- |
|  |  |  | **(1)** | **(2)** | **(3)** | **(4)** |  | **(1)** | **(2)** |  | **(1)** | **(2)** | **(3)** |  |  |
| Jung et al.^24^ | 2023 |  | **🟑** | **🟑** | **🟑** | **🟑** |  | **🟑** | - |  | **🟑** | **🟑** | **🟑** |  | 8 |
| Jung et al.^27^ | 2024 |  | **🟑** | **🟑** | **🟑** | **🟑** |  | **🟑** | - |  | **🟑** | **🟑** | **🟑** |  | 8 |
| Park et al.^30^  2023 | 2023 |  | **🟑** | **🟑** | **🟑** | **🟑** |  | **🟑** | - |  | **🟑** | **🟑** | **🟑** |  | 8 |

**Selection 0-4🟑**

(1) Representativeness of the exposed cohort.

(2) Selection of the non-exposed cohort.

(3) Ascertainment of exposure.

(4) Demonstration that the outcome of interest was not present at the start of the study.

**Comparability 0–2🟑**

Comparability of cohorts on the basis of the design or analysis

(1) Study controls for PPI-based regimens (select the most important factor)

(2) Study controls for any additional factor (This criteria could be modified to indicate a specific control for a second important factor.)

**Outcome 0–3🟑**

(1) Assessment of the outcome

(2) Was follow-up long enough for the outcomes to occur?

(3) Adequacy of follow up of the cohorts
