# Supplementary material for: Comparison of tegoprazan-based and proton pump inhibitor-based regimens for Helicobacter pylori eradication: a meta-analysis and systematic review
Source: Front Med (Lausanne). 2025 Jun 18;12:1580203. doi: 10.3389/fmed.2025.1580203 (PMC12215699; doi:10.3389/fmed.2025.1580203)
Supplement: Supplementary file 4 [file Table_2.docx]

| #1 | "Helicobacter pylori"[Mesh] |
| --- | --- |
| #2 | (((Helicobacter nemestrinae[Title/Abstract]) OR (Campylobacter pylori[Title/Abstract])) OR (Campylobacter pylori subsp. pylori[Title/Abstract])) OR (Campylobacter pyloridis[Title/Abstract]) |
| #3 | "1-(5-(2-fluorophenyl)-1-(pyridin-3-ylsulfonyl)-1H-pyrrol-3-yl)-N-methylmethanamine" [Supplementary Concept] |
| #4 | ((((( tegoprazan[Title/Abstract]) OR (TAK 438[Title/Abstract])) OR (TAK438[Title/Abstract])) OR (TAK-438[Title/Abstract])) OR (Takecab[Title/Abstract])) OR (potassium-competitive acid blocker [Title/Abstract])) OR (tegoprazan [Title/Abstract]) |
| #5 | #1 OR #2 |
| #6 | #3 OR #4 |
| #7 | #5 AND #6 |
